# Professional Esprit De Corps1Read at the annual meeting of the Medical Society of the County of Genesee.

**Published:** 1905-04

**Authors:** John W. Le Seur

**Affiliations:** Batavia, N. Y.


### Professional Esprit de Corps.[1]

By JOHN W. LE SEUR, M. D., Batavia, N. Y.

IN OBEDIENCE to your resolution passed at the last session of this society, I endeavor to present a paper. It is a difficult matter to select a subject which is characteristic of novelty and usefulness, and in presenting the topic I have chosen today I have in mind only an endeavor to unify and strengthen an organisation which I believe may be made very useful to the physicians of Genesee county. Hence, I ask you for a few minutes to consider professional esprit de corps.

When a physician takes his hard-earned diploma, opens his office and enters upon the practice of his profession, he becomes gradually, almost unconsciously, but always increasingly, an autocrat. The very work in which he is engaged, the very unusual responsibility he must assume, the necessity of taking charge of affairs and directing the actions of others, the imperative nature of his duties, the necessity for demanding, securing and insisting upon obedience to his orders,—all these and many other things combine to make him a self-sufficient, and he is sometimes led to believe, an independent member of society.

The multiplicity of his duties, the varied nature of his occupation, the incessant demand upon his skill, his intelligence, his judgment, his sympathy, make him an individual in a class alone and unlike almost every other. He is of necessity *sui generis.* The difficulty of leaving his many duties for even a single day makes a cogent and forceful reason for a limitation of the number of his meetings with his professional brethren. Today I try to bring home to you all a truth which each of you knows quite as well or better than I do—namely, that association, conference, discussion, unity of effort, persistency, unselfishness, fidelity and sympathy and thorough organisation are all necessary to the highest and best medical efforts and professional enjoyment and success.

A unified medical profession is vastly more likely to command the respect and esteem of the public than a divided and quarrelsome profession. I shall mention some reasons why I deem it important that due consideration be given to our duties to each other and to our organised societies and institutions; or, in a word, why it is important for us to cultivate esprit de corps.

First. Our effectiveness will be increased if we work unitedly. It matters not what may be the object in view, two men working together always accomplish more than two men work-

1. Read at the annual meeting of the Medical Society of the County of Genesee.

ing separately, especially if they are united in a definite purpose to achieve a worthy object. Twenty can do ten times as much as two, and vastly more than this when each one of the twenty has the sympathy and coöperation of his co-laborers. Cromwell's army of Round Heads was invincible, because every member of that army had a keen sense of his individual responsibility, of the importance of his work, and of his ability to increase the effectiveness of the whole army. When every man can feel that he has the power to accomplish much by uniting his best effort with that of his fellow-laborers, when he feels that because he can do this he ought to do it, when he sees his own advantage and the advantage of his profession in unified action, then he will be willing and anxious to do his part towards the accomplishment of whatever unified counsels seem most desirable.

Granting the advantages of unified effort and of a spirit of fraternity and love for the profession as a unity, how can we best secure these? I venture to suggest a few of the ways that have occurred to me. You will doubtless think of many and better ways.

Second. I believe that esprit de corps would be promoted by an increased devotedness to medical societies and to physicians' clubs. There is a very high authority which says, "As ye have opportunity, therefore do good unto all men, especially as be of the household of faith." I do not claim that this was written with the intention that it apply to any particular body of professional men, but I do believe that it expresses a truth, the importance of which we cannot overestimate, and that it will be well worth our while to remember it in the professional and social duties of our everyday lives as physicians. When opportunity comes to do good, financially, socially, politically, professionally, to a brother physician, it is well worth while for us to meet this opportunity not alone because it will benefit the body professionally as a whole, but because a certain reflex benefit will accrue to the individual making this effort. We must be willing to recognise the fact that some of us will excel in one department and some in another, and we should be willing to recognise and utilise the best wherever it may be found, and by so doing strengthen our medical fraternity as a whole, and give increased advantage to those who may be the recipients of our professional care.

Third. We may promote esprit de corps by the exercise of sympathy and regard for one another. It is too often the case that physicians fail to speak the kindly word, each for the other, which is so easy and so effective in securing the respect of the public for the medical profession. The lines of life do not fall to us all in equally pleasant places. Not all of us have the same measure of what men call success. Not all of us have equally pleasant places in which to toil. Some of us are greatly inconvenienced and hampered in our work by our surroundings. It sometimes occurs that those who have the hardest work and greatest privations have least of social or financial reward for their labors. Sometimes one of our professional family is so located that it is exceptionally difficult for him to meet his fellows, and he has a hard struggle against odds in his field of toil. To such a one our sympathy and cordiality, when he does attend one of our meetings, will prove wonderfully helpful. And if we find anyone of the members of our professional family burdened with a heavy load of responsibility and care, a kindly word of sympathy and cheer will be much appreciated. Each will thus make the other feel that there is pleasure as well as profit in society affiliation and work, and we may all thus promote esprit de corps.

Fourth. The spirit of the body, fraternity, good fellowship, in any organisation may be promoted by loyal support. The Genesee County Medical Society is professionally young as a distinctive body, but it is growing rapidly in members and in the scope of its usefulness. Perhaps even more gratifying than these pleasant features are the facts that with this numerical increase and enlarged sphere of activity the attendance has steadily increased, the character of the work is maintained, and last, but by no means least, the unified spirit of loyal support grows broader and deeper with each succeeding meeting of the association.

I think it has come to be true in this society that, without conference, each member may reasonably depend upon every member in Genesee county to do that which seems right and best for the interest of the profession as a whole. Every other line of intelligent activity today tends to organise with a view to increased effectiveness, and there is no reason why the medical profession should not utilise this idea for its own benefit and thus benefit its patrons. We can enter upon our work with greater satisfaction, we can secure greater effectiveness as physicians in this county when we feel that every physician legally qualified to practise, is a member of this society and is anxious to do everything he possibly can to make the society effective and helpful. It will be a source of increased satisfaction to all of us to feel that every member of this society may be counted upon for loyal support for every measure that is based on justice and equity; and this means something more than cheering for the society, something more than attending the meetings of the society, something more than helping to eat a good dinner together once a month.

It means working for the society by writing papers, by reporting cases, by making investigations, by defending the rights of physicians in state and national committees, and striving by every means within our power to attain a high standard of excellence in our local and state society work, that we may command the respect of our enemies and deserve the encomiums of our friends.

The same local support that will make our societies in the highest degree successful will make us each appreciate the other more fully, that the weaker may be benefited by the strength of the stronger, and the stronger strengthened by the sympathy of the weak; and so it may come to be true that increasing satisfaction, success and strength shall be ours by an intelligent cultivation of what, for want of a better term, I today designate esprit de corps.

We shall be better acquainted and we shall learn to depend each upon the other; we shall learn to cultivate a spirit of fraternity and friendship and thus, with every member at his best, not alone for self, but losing selfishness in our thoughtfulness for our great common cause, we shall have pride and pleasure in esprit de corps.

